# Real-world severe COVID-19 outcomes associated with use of antivirals and neutralising monoclonal antibodies in Scotland

**DOI:** 10.1038/s41533-024-00374-x

**Published:** 2024-06-28

**Authors:** Holly Tibble, Tanja Mueller, Euan Proud, Elliott Hall, Amanj Kurdi, Chris Robertson, Marion Bennie, Lana Woolford, Lynn Laidlaw, Kamil Sterniczuk, Aziz Sheikh

**Affiliations:** 1 Usher Institute, University of Edinburgh, Edinburgh, Scotland; 2 Public Health Scotland, Glasgow, Scotland; 3 Strathclyde Institute of Pharmacy and Biomedical Sciences, University of Strathclyde, Glasgow, Scotland; 4 Department of Pharmacology, College of Pharmacy, Al-Kitab University, Kirkuk, Iraq; 5 Department of Public Health Pharmacy and Management, School of Pharmacy, Sefako Makgatho Health Sciences University, Pretoria, South Africa; 6 Department of Mathematics and Statistics, University of Strathclyde, Glasgow, Scotland; 7 Patient Contributor, London, UK

**Keywords:** Therapeutics, Prognosis

## Abstract

We sought to investigate the incidence of severe COVID-19 outcomes after treatment with antivirals and neutralising monoclonal antibodies, and estimate the comparative effectiveness of treatments in community-based individuals. We conducted a retrospective cohort study investigating clinical outcomes of hospitalisation, intensive care unit admission and death, in those treated with antivirals and monoclonal antibodies for COVID-19 in Scotland between December 2021 and September 2022. We compared the effect of various treatments on the risk of severe COVID-19 outcomes, stratified by most prevalent sub-lineage at that time, and controlling for comorbidities and other patient characteristics. We identified 14,365 individuals treated for COVID-19 during our study period, some of whom were treated for multiple infections. The incidence of severe COVID-19 outcomes (inpatient admission or death) in community-treated patients (81% of all treatment episodes) was 1.2% (*n* = 137/11894, 95% CI 1.0-1.4), compared to 32.8% in those treated in hospital for acute COVID-19 (re-admissions or death; *n* = 40/122, 95% CI 25.1-41.5). For community-treated patients, there was a lower risk of severe outcomes (inpatient admission or death) in younger patients, and in those who had received three or more COVID-19 vaccinations. During the period in which BA.2 was the most prevalent sub-lineage in the UK, sotrovimab was associated with a reduced treatment effect compared to nirmaltrelvir + ritonavir. However, since BA.5 has been the most prevalent sub-lineage in the UK, both sotrovimab and nirmaltrelvir + ritonavir were associated with similarly lower incidence of severe outcomes than molnupiravir. Around 1% of those treated for COVID-19 with antivirals or neutralising monoclonal antibodies required hospital admission. During the period in which BA.5 was the prevalent sub-lineages in the UK, molnupiravir was associated with the highest incidence of severe outcomes in community-treated patients.

## Introduction

SARS-CoV-2, which causes the disease now commonly known as COVID-19, has constantly been evolving. As of December 2022, the Omicron virus variant remains the only variant classified as a ‘Variant of Concern’ (or VoC) by the World Health Organization (WHO) after Delta was de-escalated in June 2022 based on low levels of circulation. Three sub-lineages of the Omicron variant are currently classified as VoC by the European Centre for Disease Prevention and Control (ECDC), including BA.2, BA.4, and BA.5^1^.

Following encouraging clinical trial results^2,3^, UK treatment guidelines recommended use of monoclonal antibodies (mABs) tocilizumab and sarilumab, immune-modulators blocking interleukin-6 receptors; and casirivimab + imdevimab (Ronapreve) and sotrovimab (Xevudy), neutralising monoclonal antibodies (nMABs) specifically targeting spike proteins of SARS-CoV-2. While the former two are used (off-label for sarilumab) in hospitalised patients with severe disease, the latter two were approved for hospitalised and/or non-hospitalised patients with acute infection to prevent disease progression in September and December 2021, respectively^4–6^. The antiviral drug remdesivir received emergency authorisation for use in COVID-19 in the UK in May 2020; subsequently, molnupiravir (Lagevrio) and nirmaltrelvir + ritonavir (Paxlovid) were granted regulatory approval in December 2021 for the prevention of disease progression in vulnerable patients, i.e., those immunocompromised either due to underlying conditions or concurrent treatments. Finally, baricitinib (which is licensed in the UK for treatment of rheumatoid arthritis and atopic dermatitis) was added to treatment guidelines for those hospitalised with acute-severe COVID-19 in May 2022^7^. A timeline of treatment authorisation in the UK is presented in Supplementary fig. 1.

UK COVID-19 treatment guidelines have undergone frequent changes over time in line with emerging evidence^8^, mostly in relation to the potential impact of virus mutations on the effectiveness of vaccines and other therapeutic options. For instance, the treatment efficacy of casirivimab + imdevimab was found to be reduced against the Omicron variant compared to the Delta variant^9^; consequently, there is now a strong recommendation against its use from the WHO^10^. Similarly, it has been suggested that SARS-CoV-2 may develop resistance to sotrovimab due to the limited number of targets within the virus genome^9,11^, and the WHO therefore now also recommends against its use in patients with non-severe COVID-19^10^.

In addition to emerging evidence from rapid in-vitro studies^9,11–14^, several large-scale randomised controlled trials have been conducted to assess treatment efficacy of COVID-19 treatments in diverse populations with varying comorbidities and vaccination status, including the PANORAMIC^15^ and RECOVERY^7,16,17^ trials. Observational evidence is also necessary, however, to assess outcomes in patients treated for emerging sub-lineages in real-world settings, including off-label use.

Our aim was to compare the effectiveness of antivirals and nMAbs in preventing severe outcomes from COVID-19 in adult patients in Scotland, and to investigate differences in outcomes in for different treatments delivered in the community setting between variants and sub-lineages.

## Methods

### Study design

We undertook a national retrospective cohort study, comparing clinical outcomes in those treated for COVID-19 in Scotland by treatment and across time-periods when different sub-lineages were prevalent.

### Study cohort

The study cohort were all individuals treated for COVID-19 between December 21, 2021, and September 26, 2022. For this analysis, the drugs of interest were molnupiravir, nirmaltrelvir + ritonavir, remdesivir, sotrovimab, sarilumab, and tocilizumab. Casirivimab + imdevimab was also licensed for use in Scotland at the same time, but it was excluded from this analysis due to low uptake across the country following reports of lack of efficacy against Omicron sub-lineages^14^. Baricitinib was also excluded as it was not captured in the study data sources^18^.

As a person could be treated for multiple infections, records were separated into estimated infection-treatment episodes of at most 40 days in duration. Within an episode, an individual may still have been treated with multiple medications, either sequentially if they did not respond to the first treatment option or at the same time if they were at especially high risk and/or had severe symptom presentation. Cases in which the first administration or prescription of each therapy were initiated within three days of each other were classified as combination treatments; all other episodes were categorised by the first therapy given.

Patients were sub-grouped according to whether they were treated in the community or in a hospital setting. Group 1 patients were defined as those treated for acute COVID-19, during a hospital admission of at least one night’s duration, with COVID-19 as the primary cause. Group 2 patients were defined as those treated during a hospital admission, of at least one night’s duration, without COVID-19 as the primary cause. Group 3 patients were treated outside of an acute hospital admission (or during day visits, for medication administration). Finally, Group 4 patients were those treated during a currently uncoded hospital admission of at least one night’s duration, and thus with insufficient data to classify them into Group 1 or 2. As sarilumab and tocilizumab were only indicated for patients with severe acute COVID-19 requiring admission, treatment(s) for which an admission could not be identified were excluded, as this was a likely case of treatment in a specialist inpatient unit (such as cancer or maternity departments) for which no data were available.

### Data sources

Information regarding the prescription/administration of any of the drugs of interest was captured from multiple data sources, at the Health Board (Scottish regional authority for health care service delivery) level^18^. First, data were purposely collected through spreadsheets (henceforth Public Health Scotland Order, or PHSO), with weekly updates provided directly by the regional Health Boards (who manage their territories hospitals, district nursing services and healthcare planning). At least one report was received from 13/14 Health Boards (Supplementary Fig. 2) between December 21, 2021, and September 26, 2022 (with different end points per Health Board in the PHSO data, as listed in Supplementary Methods). Second, data on drug exposure were available for six Health Boards from the Hospital Electronic Prescribing and Medicines Administration System (HEPMA). Finally, data for one Health Board (NHS Lothian) were extracted from the prescribing information system (PIS)^19^. For all data sources, prescriptions had to be associated with a valid Community Health Index (CHI; Scotland’s unique patient identifier number) number to enable linkage to other sources; as such, 79% of all prescriptions dispensed were retained for analysis.

We used data from the Scotland-wide Early Pandemic Evaluation and Enhanced Surveillance of COVID-19 (EAVE II) platform^20,21^, as described in Supplementary Methods. Comorbidities were estimated from inpatient admissions and medical procedures, and primary care consultations and prescriptions, with a five-year look-back period. Four sources were used to estimate the incidence of outcomes.

### Study outcomes

Clinical outcomes estimated were inpatient admissions (and re-admissions) of at least one night’s duration, ICU admissions (of any duration), and deaths, both all-cause and specifically for COVID-19, within 28 days of treatment initiation. The incidence risk of each outcome was estimated using the denominator of those with at least 28 days of follow-up in the relevant data source, or an outcome within 28 days. As an exception, however, for COVID-19 inpatient admissions, an extended minimum follow-up period was used of 42 days (28 days x1.5). The outcome was still censored at 28 days, but only those with an event within 28 days or no event within 42 days were included. This was due to the lag in SMR01 reporting of approximately 6 weeks, based on discharge date and the time required for clinical coding to take place. This more stringent follow-up requirement reduced the risk of classifying someone as having no outcome when it was simply yet to be reported, but without excluding valid data, particularly on more recently emerging variants and sub-lineages. Additionally, those in Groups 1, 2 and 4 who were yet to be discharged from hospital within 28 days of treatment initiation were excluded from hospital admission analyses. Finally, we estimated any acute COVID-19 event as any inpatient hospital admission, ICU admission, or death within 28 days.

### Confounders

The high-risk conditions estimated were chemotherapy, radiotherapy, blood cancer, respiratory cancer, cirrhosis, chronic kidney disease (CKD stage 3 + ), prescription of immunosuppressants, a neurological condition (Parkinson’s disease, motor neurone disease, multiple sclerosis or cerebral palsy), rheumatoid arthritis or systemic lupus erythematosus, a solid organ transplant, a stem cell transplant, a bone marrow transplant, HIV/AIDS, sickle cell disease, Down’s syndrome, and splenectomy^22^.

Patient demographic data were extracted from primary care registries: age, sex, Scottish Index of Multiple Deprivation (SIMD), and the Urban-Rurality index. The number of COVID-19 vaccinations prior to treatment was extracted. Finally, the first positive reverse-transcription polymerase chain reaction (RT-PCR) test, lateral flow test (LFT), or inpatient admission with primary cause as COVID-19 in the 28 days preceding treatment initiation was selected as the first date of diagnosis to enable the time between diagnosis and treatment to be estimated.

### Statistical analysis

Univarible logistic regression was used to assess risk factors (high-risk comorbidities and patient characteristics) for severe COVID-19 outcomes (hospital admissions, ICU admissions, and death) within 28 days of treatment initiation, in Group 3 patients. A stratified logistic regression was also conducted, to compare the adjusted odds ratio (aOR) of severe COVID-19 outcomes within 28 days of treatment initiation across time periods according to the most prevalent COVID-19 sub-lineage, in Group 3 patients treated with sotrovimab, nirmaltrelvir + ritonavir, or molnupiravir.

### Patient and public involvement

Patient and public contributors were involved in the design and interpretation of this study. Details of their involvement are presented in Supplementary Notes.

### Reporting guidelines

The Strengthening the Reporting of Observational Studies in Epidemiology (STROBE) checklist for cohort studies is presented in Supplementary Table 1.

### Role of the funding source

The funders of the study had no role in study design, data collection, data analysis, data interpretation, or writing of the report.

## Results

### Baseline characteristics

**Fig. 1:**
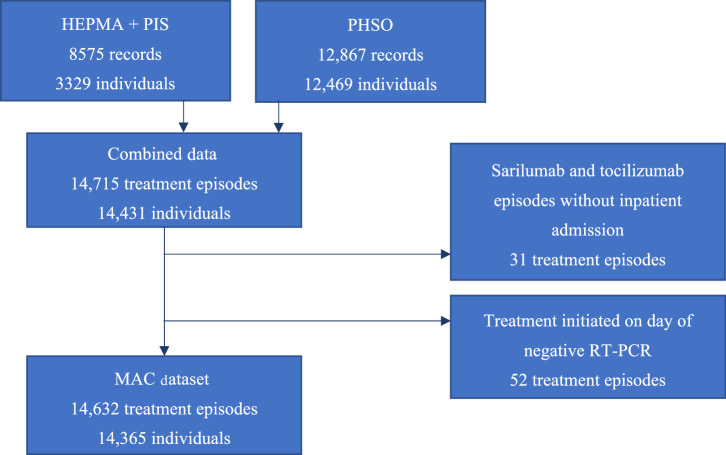
Patient Flow Diagram. 8575 records were identified through the HEPMA and PIS datasets, and 12,867 through the manual health board reporting. After removing duplicates, 14,715 records remained. After exclusions, there were 14.632 treatment episodes for analysis.

From the combined treatment data sources, 14,715 treatment episodes were identified for 14,431 individuals (Fig. 1). There were 31 treatment episodes for sarilumab and tocilizumab that were excluded as a corresponding admission could not be identified, as well as 52 episodes in which treatment was initiated on the same day as a negative RT-PCR test (0.35% of all episodes). As such, there were a final total of 14,632 treatment episodes included, for 14,365 individuals.

1,232 (8.4%) treatment episodes were during acute COVID-19 inpatient admissions (Group 1), 1,467 (10.0%) during admissions with hospital onset or concurrent COVID-19 (Group 2), 11,894 (81.3%) outside of an admission (treated in the community with non-severe COVID-19; Group 3) and 39 (0.3%) during admissions which had not yet been coded (Group 4). Table 1 shows this broken down further by treatment and patient demographics, and Supplementary Table 2 shows the patient comorbidities for Group 3 patients treated with molnupiravir, nirmaltrelvir + ritonavir, or sotrovimab.

**Table 1:** Patient characteristics for treatment episodes, by healthcare setting.

	Healthcare setting: number (episodes) treated (column percent)			
	Hospitalised for acute COVID-19 (Group 1)	Hospital-onset or concurrent COVID-19 (group 2)	Non-hospitalised (group 3)	Uncoded hospitalisation (group 4)
	*N* = 1232	*N* = 1467	*N* = 11894	*N* = 39
Medication				
Combination	126 (10.2%)	73 (5.0%)	47 (0.4%)	≤5 ( < 12.8%)
Molnupiravir	12 (1.0%)	9 (0.6%)	2910 (24.5%)	7 (17.9%)
Nirmaltrelvir + Ritonavir	124 (10.1%)	371 (25.3%)	5688 (47.8%)	9 (23.1%)
Remdesivir	391 (31.7%)	529 (36.1%)	20 (0.2%)	9 (23.1%)
Sarilumab	267 (21.7%)	26 (1.8%)	0	≤5 ( < 12.8%)
Sotrovimab	129 (10.5%)	352 (24.0%)	3229 (27.1%)	12 (30.8%)
Tocilizumab	183 (14.9%)	107 (7.3%)	0	≤5 ( < 12.8%)
Age group				
0–17 or Unknown	16 (1.3%)	13 (0.9%)	168 (1.4%)	≤5 ( < 12.8%)
18–40	105 (8.5%)	73 (5.0%)	2492 (21.0%)	7 (17.9%)
41–60	332 (26.9%)	303 (20.7%)	5097 (42.9%)	≤5 ( < 12.8%)
61–75	439 (35.6%)	541 (36.9%)	3378 (28.4%)	10 (25.6%)
76+	340 (27.6%)	537 (36.6%)	759 (6.4%)	16 (41.0%)
Sex				
Male	664 (53.9%)	756 (51.5%)	4647 (39.1%)	14 (35.9%)
Female	562 (45.6%)	709 (48.3%)	7168 (60.3%)	24 (61.5%)
Unknown/Other	6 (0.5%)	≤5 (≤0.3%)	79 (0.7%)	≤5 ( < 12.8%)
Vaccinations				
0–2	459 (37.3%)	312 (21.3%)	526 (4.4%)	11 (28.2%)
3	538 (43.7%)	864 (58.9%)	6316 (53.1%)	16 (41.0%)
4+	235 (19.1%)	291 (19.8%)	5052 (42.5%)	12 (30.8%)
Comorbidities				
Chemotherapy	139 (11.3%)	192 (13.1%)	1540 (12.9%)	10 (25.6%)
Blood Cancer	93 (7.5%)	68 (4.6%)	998 (8.4%)	≤5 ( < 12.8%)
Bone Marrow Transplant	≤5 (≤0.4%)	≤5 (≤0.3%)	7/11894 (0.1%)	≤5 ( < 12.8%)
Cirrhosis	14 (1.1%)	38 (2.6%)	250 (2.1%)	≤5 ( < 12.8%)
Chronic Kidney Disease (Stage 3 + )	240 (19.5%)	348 (23.7%)	1715 (14.4%)	12 (30.8%)
Down Syndrome	≤5 (≤0.4%)	≤5 (≤0.3%)	109/11894 (0.9%)	≤5 ( < 12.8%)
HIV/AIDS	≤5 (≤0.4%)	≤5 (≤0.3%)	97/11894 (0.8%)	≤5 ( < 12.8%)
Immunosuppressants Prescribed	121 (9.8%)	94 (6.4%)	2054 (17.3%)	8 (20.5%)
Neurological Condition	26 (2.1%)	31 (2.1%)	1846 (15.5%)	8 (20.5%)
Radiotherapy	10 (0.8%)	48 (3.3%)	107 (0.9%)	≤5 ( < 12.8%)
Rheumatoid Arthritis or Systemic Lupus Erythematosus	84 (6.8%)	94 (6.4%)	2208 (18.6%)	≤5 ( < 12.8%)
Respiratory Cancer	9 (0.7%)	18 (1.2%)	72 (0.6%)	≤5 ( < 12.8%)
Sickle Cell Disease	≤5 (≤0.4%)	≤5 (≤0.3%)	11/11894 (0.1%)	≤5 ( < 12.8%)
Solid Organ Transplant	217 (17.6%)	267 (18.2%)	2002 (16.8%)	13 (33.3%)
Stem Cell Transplant	12 (1.0%)	7 (0.5%)	70 (0.6%)	≤5 ( < 12.8%)

Splenectomy was excluded from analyses as all healthcare setting groups were required to suppress low numbers to protect patient confidentiality.

**Fig. 2:**
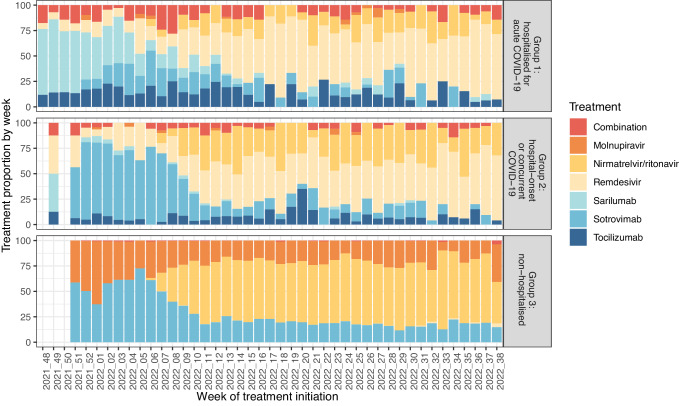
Treatment by healthcare setting (groups 1–3) and week of initiation. Notes: Weeks with fewer than five patients treated (in a single healthcare setting) were omitted from the graph for patient confidentiality. Data for Group 3 patients previously reported in Tibble et al. 2022^18^.

Figure 2 shows the initiated treatment proportions by week and healthcare setting (excluding Group 4: those currently uncoded inpatient treatments). There were a smaller proportion of patients treated with sarilumab since March 2022; remdesivir was the most common treatment in Groups 1 and 2, and nirmaltrelvir + ritonavir in Group 3. Supplementary Table 3 shows the concordance between the sequencing results and the most prevalent sub-lineage at that time, and the number of patients treated with each treatment by most prevalent sub-lineage. The positive predictive value (percent of sequenced individuals the most dominant sub-lineage in that period) was 71.8% in the BA.1 period, 89.2% in the BA.2 period, and 73.2% in the BA.5 period (Supplementary Table 4).

### Incidence of outcomes

Overall, there were 12,417 treatment episodes with 42 days of follow-up in SMR01 after treatment initiation or an admission within 28 days (in those discharged from their index admission if in Groups 1, 2, or 4). Of these, 193 (1.6%) were admitted to hospital with a primary diagnosis of COVID-19 within 28 days of treatment initiation (or readmitted, for those treated initially within the hospital setting; Table 2). 0.5% of patients had COVID-19 ICU admissions within 28 days, and 1.7% died of COVID-19. For all outcomes, the risk was lowest in Group 3 and highest in Group 1. For all-cause mortality, the risk was similar between those treated in Groups 1 and 2.

**Table 2:** Clinical outcomes within 28 days of treatment initiation, overall and by healthcare setting.

Outcome (within 28 days)	Number of episodes with outcome / Number of eligible episodes (Percent with outcome) [95% Confidence Intervals]			
	Overall	Hospitalised for acute COVID-19 (Group 1)	Hospital-onset or concurrent COVID-19 (Group 2)	Non-hospitalised (Group 3)
Any COVID-19 event	193/12417 (1.6%) [1.4–1.8%]	40/122 (32.8%) [25.1–41.5%]	13/380 (3.4%) [2.0–5.8%]	137/11894 (1.2%) [1.0–1.4%]
COVID-19 hospital admission	165/12417 (1.3%) [1.1–1.5%]	25/122 (20.5%) [14.3–28.5%]	10/380 (2.6%) [1.4–4.8%]	127/11894 (1.1%) [0.9–1.3%]
All-cause hospital admission	820/12711 (6.5%) [6.0–6.9%]	150/247 (60.7%) [54.5–66.6%]	184/545 (33.8%) [29.9–37.8%]	480/11894 (4.0%) [3.7–4.4%]
COVID-19 ICU admission	80/14632 (0.5%) [0.4–0.7%]	60/1232 (4.9%) [3.8–6.2%]	7/1467 (0.5%) [0.2–1.0%]	13/11894 (0.1%) [0.1–0.2%]
All-cause ICU admission	162/14632 (1.1%) [0.9–1.3%]	80/1232 (6.5%) [5.2–8.0%]	45/1467 (3.1%) [2.3–4.1%]	35/11894 (0.3%) [0.2–0.4%]
COVID-19 death	245/14632 (1.7%) [1.5–1.9%]	162/1232 (13.1%) [11.4–15.2%]	66/1467 (4.5%) [3.6–5.7%]	14/11894 (0.1%) [0.1–0.2%]
All-cause death	462/14632 (3.2%) [2.9–3.5%]	196/1232 (15.9%) [14.0–18.1%]	222/1467 (15.1%) [13.4–17.1%]	41/11894 (0.3%) [0.3–0.5%]

The denominator population for the outcome ‘any COVID-19 event was those with 42 days of follow-up in SMR01, as with the ‘COVID-19 hospital admission’ outcome.

The denominator population for Groups 1, 2, and 4 (included in the overall column) included only those who had been discharged within 28 days of their diagnosis.

Group 4 has been omitted (although still included in the overall column) due to limited inference with small numbers.

Table 3 shows the proportion of patients treated for non-severe COVID-19 in the community with severe outcomes (hospitalisation and death, both for COVID-19 and all-cause) within 28 days of treatment initiation. Nirmaltrelvir + ritonavir-treated patients had the lowest incidence of all outcomes except COVID-19 mortality (lowest for sotrovimab). Those treated with a combination of therapies (likely to be the most high-risk patients, or those with the most severe presentation) had the highest incidence of all outcomes (excluding for remdesivir, for which inference is extremely limited due to very small numbers). Neither ICU-based outcomes nor outcomes for those treated in the hospital setting could be reported stratified by treatment due to small numbers.

**Table 3:** Clinical outcomes within 28 days of treatment initiation by treatment in those treated in the community with non-severe COVID-19 (Group 3).

Medication	Number of episodes with outcome / Number of eligible episodes (Percent with outcome) [95% Confidence Intervals]				
	Any COVID-19 event	COVID-19 hospital admission	All-cause hospital admission	COVID-19 death	All-cause death
Combination	10/47 (21.3%) [12.0–34.9%]	9/47 (19.1%) [10.4–32.5%]	12/47 (25.5%) [15.3–39.5%]	2/47 (4.3%) [1.2–14.2%]	2/47 (4.3%) [1.2–14.2%]
Molnupiravir	65/2910 (2.2%) [1.8–2.8%]	62/2910 (2.1%) [1.7–2.7%]	164/2910 (5.6%) [4.9–6.5%]	7/2910 (0.2%) [0.1–0.5%]	23/2910 (0.8%) [0.5–1.2%]
Nirmaltrelvir + Ritonavir	26/5688 (0.5%) [0.3–0.7%]	22/5688 (0.4%) [0.3–0.6%]	145/5688 (2.5%) [2.2–3.0%]	4/5688 (0.1%) [<0.1–0.2%]	6/5688 (0.1%) [<0.1–0.2%]
Remdesivir	<5/20	<5/20	8/20 (40.0%) [21.9–61.3%]	0/20	3/20 (15.0%) [5.2–36.0%]
Sotrovimab	33/3229 (1.0%) [0.7–1.4%]	32/3229 (1.0%) [0.7–1.4%]	151/3229 (4.7%) [4.0–5.5%]	1/3229 (<0.1%) [<0.1–0.2%]	7/3229 (0.2%) [0.1–0.4%]

### Factors associated with COVID-19 hospital admission

In univariable analyses, having been diagnosed with blood cancer or CKD, or having had chemotherapy or a solid organ transplant were all associated with higher odds of COVID-19 inpatient admission, while diagnoses of neurological conditions, rheumatoid arthritis, or systemic lupus erythematosus were associated with lower odds (Table 4) compared to other treated COVID-19 patients without record of these comorbidities. Those aged over 60 were significantly more likely to have an admission than those aged 18-40, and men were more likely to have an admission than women. Those with 3 or more COVID-19 vaccinations were significantly less likely to have a COVID-19 admission than those with 2 or fewer vaccinations.

**Table 4:** Univariable logistic regression analyses for odds ratio of having any COVID-19 outcome within 28 days of treatment initiation, for those treated in the community with non-severe COVID-19.

Risk factor	*n*	Odds ratio (95% confidence interval)	*p* value
Comorbidities			
Chemotherapy	1540	1.585 (1.030–2.437)	0.036
Blood Cancer	998	2.474 (1.596–3.835)	<0.001
Cirrhosis	250	0.340 (0.047–2.440)	0.283
Chronic Kidney Disease (Stage 3 + )	1715	2.394 (1.646–3.482)	<0.001
Immunosuppressants Prescribed	2054	1.467 (0.985–2.186)	0.059
Neurological Condition	1846	0.472 (0.254–0.876)	0.017
Rheumatoid Arthritis or Systemic Lupus Erythematosus	2208	0.342 (0.180–0.653)	0.001
Solid Organ Transplant	2002	1.844 (1.261–2.697)	0.002
No identified comorbidities	3344	0.777 (0.522–1.157)	0.214
Urban Rurality			
Urban Area	7079	{ref}	
Small Towns	1100	1.061 (0.601–1.874)	0.839
Rural Area	1687	1.037 (0.641–1.677)	0.882
Missing	2028	0.696 (0.412–1.174)	0.174
Age group			
18–40	2492	{ref}	
41–60	5097	1.584 (0.849–2.957)	0.148
61–75	3378	3.273 (1.788–5.991)	<0.001
76+	759	5.959 (3.004–11.822)	<0.001
Sex			
Female	7168	{ref}	
Male	4647	1.671 (1.190–2.347)	0.003
SIMD Quintile	0.819 (0.626 – 1.070)	0.114	
Vaccinations			
0–2	526	{ref}	
3	6316	0.410 (0.229–0.733)	0.003
4+	5052	0.338 (0.214–0.704)	0.002
Time between diagnosis and treatment			
Same Day	799	{ref}	
Next Day	1371	0.541 (0.266–1.101)	0.090
2–3 Days	1905	0.519 (0.268–1.007)	0.053
4–28 Days	131	1.147 (0.330–3.992)	0.829
No Test Identified	7688	0.534 (0.311–0.917)	0.023

The following high-risk categorised comorbidities were excluded from analyses as there were fewer than 150 total cases: Bone Marrow Transplant, HIV/AIDS, Respiratory Cancer, Sickle Cell Disease, Down Syndrome, Radiotherapy, Splenectomy, and Stem Cell Transplant. Those with missing age (*n* = 79), sex (*n* = 79), and SIMD quintile (*n* = 100), as well as those aged under 18 (*n* = 89), were excluded.

In multivariable analyses, stratified into time-periods by most prevalent sub-lineage, molnupiravir treatment was consistently associated with the highest proportion of severe outcomes in Group 3 patients (Table 5). While BA.2 was most prevalent sub-lineage, nirmaltrelvir + ritonavir treatment was associated with the lowest incidence of severe outcomes, and sotrovimab was associated with 5.7 times higher aOR of severe outcomes than nirmaltrelvir + ritonavir. However, for sub-lineages BA.1. and BA.5, sotrovimab treated patient had the lowest incidence of severe outcomes (very similar incidence to nirmaltrelvir + ritonavir in BA.5 period). Having been diagnosed with blood cancer was only found to significantly increase risk of COVID-19 events in the period in which BA.1 was the most prevalent sub-lineage of the virus (aOR = 2.801, 95% CI 1.052 – 7.459). Similarly, diagnoses of neurological conditions were only associated with a lower risk of events in the BA.5 period (aOR = 0.114, 95% CI 0.015 – 0.844). There was a consistent trend across time periods that older age groups were more at risk, and that ≥3 vaccinations decreased risk. There was no observed association between sex and event risk in these adjusted analyses.

**Table 5:** Multivariable logistic regression analyses for odds ratio of having any COVID-19 outcome within 28 days of treatment initiation, for those treated in the community with non-severe COVID-19, stratified by most prevalent sub-lineage at time of diagnosis.

Risk factor / most prevalent sub-lineage in period	December 21, 2021, to February 28, 2022 (BA.1)	March 1, 2022, to May 31, 2022 (BA.2)	June 1, 2022, to September 26, 2022 (BA.5)
	Odds Ratio (95% Confidence Interval)		
Medication			
Nirmaltrelvir + Ritonavir vs Molnupiravir	0.491 (0.110–2.192)	**0.129 (0.050–0.331)**	**0.379 (0.197–0.730)**
Sotrovimab vs Molnupiravir	**0.376 (0.166–0.851)**	0.601 (0.294–1.225)	**0.396 (0.181–0.865)**
Nirmaltrelvir + Ritonavir vs Sotrovimab	1.036 (0.273–6.243)	**0.215 (0.078–0.593)**	0.958 (0.401–2.289)
Comorbidities			
Blood Cancer	**2.801 (1.052–7.459)**	2.179 (0.904–5.253)	1.499 (0.590–3.405)
Chemotherapy	N/A	0.718 (0.170–3.022)	0.427 (0.144–1.902)
Chronic Kidney Disease (Stage 3 + )	2.089 (0.810–5.385)	1.068 (0.483–2.261)	1.132 (0.579–3.405)
Immunosuppressants Prescribed	1.131 (0.383–3.339)	1.568 (0.597–4.117)	2.089 (0.897–4.250)
Neurological Condition	1.002 (0.274–3.666)	1.997 (0.690–5.781)	**0.114 (0.015–0.844)**
Rheumatoid Arthritis or Systemic Lupus Erythematosus	0.215 (0.028–1. 628)	1.095 (0.407–2.944)	0.515 (0.183–1.496)
Solid Organ Transplant	1.339 (0.556–3.224)	2.429 (0.734–8.038)	1.824 (0.625–4.680)
Age Group			
18–40	{ref}		
41–60	0.727 (0.245–2.154)	1.866 (0.517–6.738)	3.963 (0.912–17.218)
61–75	1.617 (0.544–4.809)	3.496 (0.977–12.507)	**5.908 (1.351–25.833)**
76+	1.733 (0.396–7.578)	**5.644 (1.356–23.500)**	**13.709 (3.172–69.547)**
Sex			
Female	{ref}		
Male	1.678 (0.779–3.615)	0.901 (0.469–1.733)	1.453 (0.829–2.548)
Vaccinations			
0–2	{ref}		
3	0.449 (0.126–1.607)	0.316 (0.088–1.137)	0.428 (0.162–1.127)
4+	0.498 (0.113–2.197)	**0.266 (0.071–0.992)**	**0.115 (0.060–0.401)**

Those aged under 18 or with missing age or sex were excluded. Chemotherapy was excluded from the multivariable analysis in the BA.1 period due to poor model fit (coefficient > 1000). Statistically significant odds ratios are highlighted in bold font.

## Discussion

81% of treatment episodes were initiated in the outpatient or community setting (Group 3), and of these 1.1% were subsequently admitted to hospital for COVID-19 within 28 days. During the period in which BA.5 and BA.1 were the most prevalent sub-lineages in the UK, sotrovimab and nirmaltrelvir + ritonavir were associated with the lowest incidence of severe outcomes in community-treated patients. While BA.2 was most prevalent, nirmaltrelvir + ritonavir treatment was associated with the lowest incidence.

In univariable analyses in Group 3 patients, having fewer than three COVID-19 vaccinations, having been diagnosed with blood cancer or chronic kidney disease, or having had chemotherapy or a solid organ transplant (compared to other treated patients without such diagnoses) were all associated with higher odds of subsequent COVID-19 inpatient admission. Adjusted analyses were conducted, stratified by time-period according to most prevalent sub-lineage.

As shown in Fig. 2, the proportions of patients allocated to each treatment changed over time. However, it is also likely that the characteristics of patients treated with each therapeutic have changed too, in lines with changing guidelines and emerging evidence. For example, in-vitro evidence suggested that sotrovimab was less effective against early Omicron sub-lineages than against Delta variants^9^ (whereas there was no evidence as such for anti-virals)^12^, which resulted in WHO strongly recommending against sotrovimab treatment in September 2022. In line with this, we observed a reduced treatment effect compared to molnupiravir during the period in which BA.2 was most prevalent in the UK, however while BA.5 has been the most prevalent sub-lineage, sotrovimab was associated with the lowest odds of acute COVID-19 outcomes.

The EPIC-HR trial (Evaluation of Protease Inhibition for COVID-19 in High-Risk Patients) evaluated the safety and efficacy of nirmaltrelvir + ritonavir in group 3 high-risk and unvaccinated adults with symptomatic COVID-19^23^. It found a relative risk reduction of 89.1% for the incidence of COVID-19-related hospitalisations or death by day 28. There were no deaths in their treated group, but 0.8% had a COVID-19-related hospitalisation. This was twice as high as the proportion observed herein (0.4%, 95% CI 0.3–0.6), however in our population, which made no exclusions based on vaccination status, we found a substantially increased risk in the sub-optimally vaccinated.

A study in Group 3 BA.2 patients in Hong Kong treated with nirmaltrelvir + ritonavir, molnupiravir, or placebo found that molnupiravir reduced risk of death by 24% (no reduction in hospitalisation risk), and nirmaltrelvir + ritonavir reduced risk of hospitalisation by 24% (95% CI 14-33), compared to the control arm^24^. We similarly observed that a higher proportion of Group 3 patients were hospitalised for COVID-19 in the molnupiravir treated cohorts (2.1%, 95% CI 1.7–2.7) compared to the nirmaltrelvir + ritonavir (0.4%, 95% CI 0.3–0.6) and sotrovimab (1.0%, 95% CI 0.7–1.4) treated cohorts. The UK PANORAMIC trial also reported that molnupiravir did not significantly reduce the (already low) rate of hospitalisations and deaths among high-risk Group 3 patients^15^, aligning with other real-world observational studies^25–27^.

A study in England conducted a comparative effectiveness study on molnupiravir and sotrovimab in community-treated patients in the BA.1 period, and found that sotrovimab use was associated with a longer time to COVID-19 inpatient admission or death, with a hazard ratio of 0.47 (95% CI 0.30–0.76)^28^. This was similar to our observed odds ratio of 0.38 (95% CI 0.10–0.81). Similarly, in their exploratory analyses in the BA.2 period, they observed a hazard ratio of 0.44 (95% CI 0.27–0.71), compared to our odds ratio of 0.60 (95% CI 0.29–1.33). They did not include nirmaltrelvir + ritonavir in their analyses.

Observational studies provide vital insights into real-world treatment outcomes, including from off-label use and in extremely ill patients who might be excluded from conventional studies. Additionally, they enable analysis of larger numbers of patients, which may help to identify small effects and interactions for further testing, in a controlled environment, to help inform a precision medicine approach to treatment allocation.

This study captured treatment outcomes across the whole of Scotland and was able to link to many key health datasets, to provide a rich multi-dimensional longitudinal patient dataset. With weekly updates being received from many Health Boards, and other routine data sources being updated daily, we were also able to monitor trends in real-time, and make rapid reports to Health Boards and decision makers.

Despite this, we are not able to provide a comparison to untreated patients, as no data were available on COVID-19 positive, high-risk patients who were either not symptomatic, had rapidly improving symptoms, did not report a positive LFT, or declined to be treated. As such, there is too much unmeasured confounding to produce a reliable and meaningful estimate of improvement in clinical outcomes. Furthermore, the exact date of symptom onset was not known. This information is particularly pertinent for those treated with antivirals, for which the effectiveness wanes substantially the longer the gap between onset and administration. Those who were diagnosed or admitted longer after their symptom onset may have been less likely to have been treated with antivirals for this reason.

There are three possible sources of bias in this study. Firstly, those admitted to hospital within the last 42 days (6 weeks) of the study period were not included in the admissions outcome analyses (or indeed the composite ‘any event’ outcome), due to the time required for admissions to be clinically coded and added to the dataset. Secondly, those treated in the hospital setting who were yet to be discharged from hospital within 28 days of treatment initiation were excluded from hospital admission analyses, as it was not possible for them to be readmitted in this time. Finally, no data on treatment allocation rationale was available, and as such there may be some treatment by indication bias which is not fully controlled for in the multivariable analysis, despite the inclusion of the high-risk comorbidities.

An estimated 80% of high-risk, COVID-19 diagnosed adults with more than three vaccinations against SARS-CoV-2 were referred for treatment in Scotland^18^. Our analysis shows that in Group 3 (community-treated) patients, compared to those with fewer than 3 vaccinations, these patients had an aOR of 0.12 – 0.50 for acute COVID-19 events within 28 days of treatment initiation. This reinforces the importance of high-risk individuals retaining immunity through offered boosters even where antiviral/nMAB treatments are available.

Herein, we identified that 33% of hospital-treated patients (Groups 1, 2, and 4) were aged over 75, compared to only 6% of Group 3 patients. Our previous study identified that early (Group 3) treatment proportions in older high-risk COVID-19 diagnosed adults were lower than in the younger adults^18^. However, in Group 3 patients during the period in which BA.5 has been the most prevalent sub-lineage, we also identified 13.7 times higher aOR of acute COVID-19 event within 28 days compared to 18-40 s. Similarly, in this period, having been prescribed an immunosuppressant or having had a solid organ transplant emerged as the highest risk comorbidities for odds of acute COVID-19 event. It should be considered whether these patients should be prioritised for early treatment, and perhaps monitored to identify whether further intervention is required.

In COVID-19 patients treated in the community setting, within 28 days of treatment 1% were hospitalised for their symptoms and 0.1% died. Outcomes were worse for those treated during an acute COVID-19 inpatient admission, and for those with suboptimal COVID-19 vaccination. During the period in which BA.5 and BA.1 were the most prevalent sub-lineages in the UK, sotrovimab was associated with the lowest incidence of severe outcomes in community-treated patients. While BA.2 was most prevalent, nirmaltrelvir + ritonavir treatment was associated with the lowest incidence.

### Data sharing

The data are stored in the Public Health Scotland TRE. To access these individual-level, confidential healthcare data, researchers will need to apply to HSC-PBPP (https://www.informationgovernance.scot.nhs.uk/pbpphsc/).

### Supplementary information

Supplementary Material

## Data Availability

The study data are held by the National Services Scotland electronic Data Research and Innovation Service (eDRIS) in the National Safe Haven. Restrictions apply to the availability of these data, which were used under license for the current study, and so are not publicly available. Data would be made available from a reasonable request to phs.edris@phs.scot [study ID: 2223-0033].

## Code availability

Analysis code, written in the R language, is available at https://github.com/EAVE-II/treatment-outcomes.
