# Supplementary material for: Real-world severe COVID-19 outcomes associated with use of antivirals and neutralising monoclonal antibodies in Scotland
Source: NPJ Prim Care Respir Med. 2024 Jun 28;34:17. doi: 10.1038/s41533-024-00374-x (PMC11213868; doi:10.1038/s41533-024-00374-x)
Supplement: Supplementary file 1 — Supplementary Material [file 41533_2024_374_MOESM1_ESM.docx]

Supplementary Figure 1: Timeline of UK Licensing Dates for COVID-19 Therapeutics


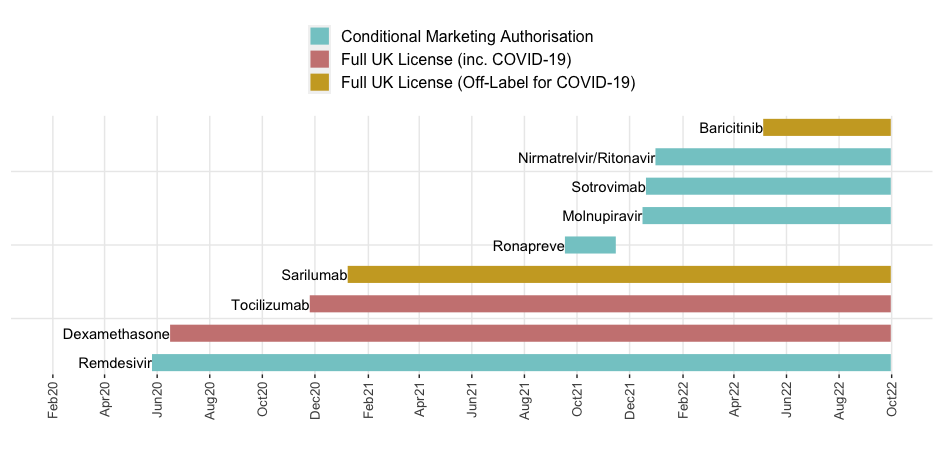


Supplementary Figure 2: Map of Drug Exposure Data Source by the 14 Scottish Health Board


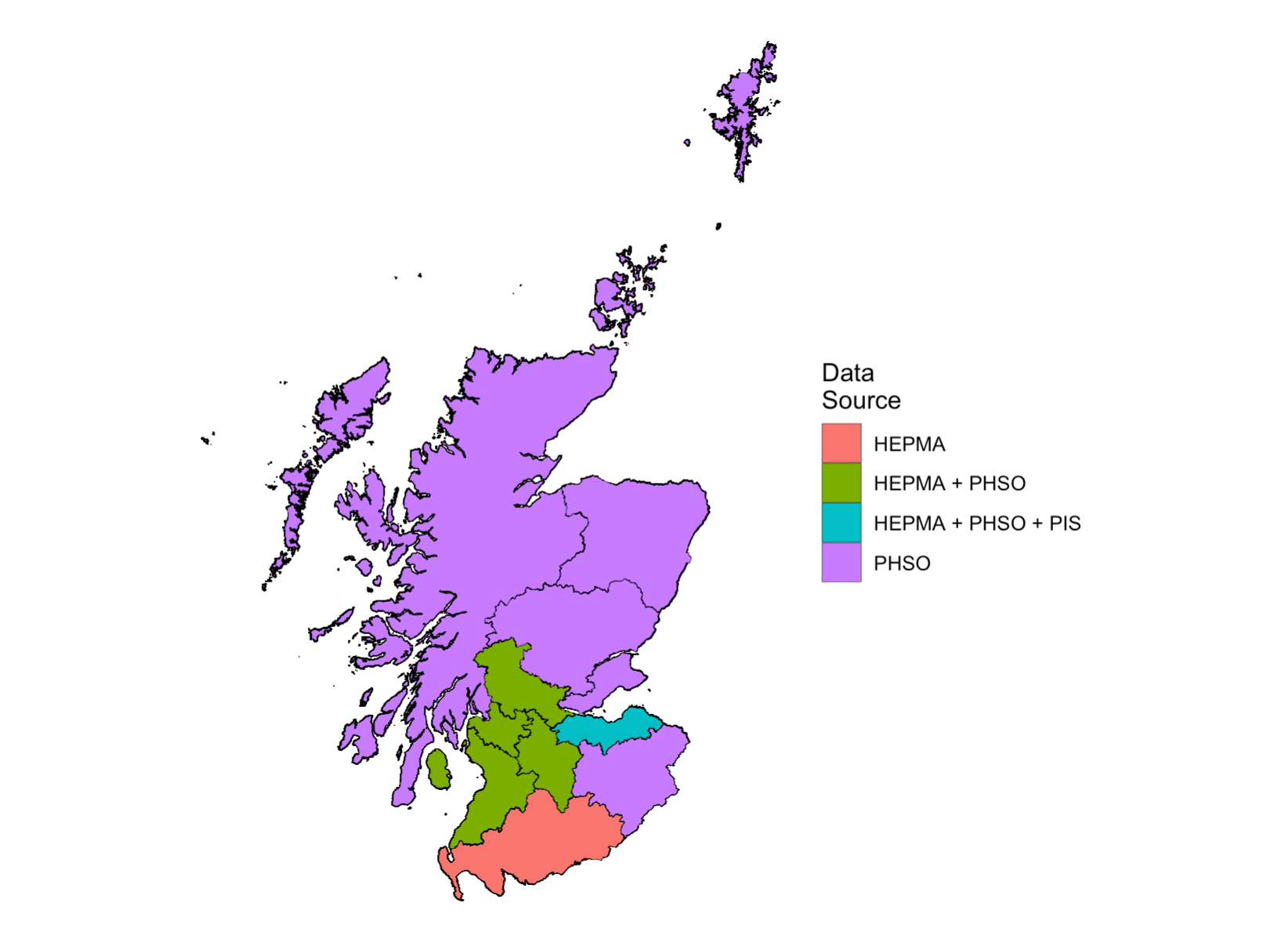


Notes: HEPMA = Hospital Electronic Prescribing and Medicines Administration System, PHSO = Public Health Scotland Order (weekly excel spreadsheets), PIS = Prescribing Information System (primary care prescribing data).

Supplementary Methods: Data Sources

Dates of Last Treatment Record per Health Board in the manually submitted spreadsheets

| Health Board | Date of Last Recorded Treatment (2022) |
| --- | --- |
| Ayrshire & Arran | September 18^th^ |
| Borders | September 22^nd^ |
| Fife | August 30^th^ |
| Forth Valley | August 13^th^ |
| Grampian | April 21^st^ |
| Greater Glasgow & Clyde | August 8^th^ |
| Highland | August 15^th^ |
| Lanarkshire | September 5^th^ |
| Lothian | September 19^th^ |
| Orkney | August 28^th^ |
| Shetland | September 7th |
| Tayside | September 23^rd^ |
| Western Isles | September 5^th^ |

Data sources from within the EAVE II platform were the Scottish Morbidity Records inpatient dataset (SMR01, data available up to April 10, 2023), Rapid Preliminary Inpatient Data (RAPID: April 17, 2023), Scottish Intensive Care Society Audit Group (SICSAG) episode-level Intensive Care Unit (ICU) admissions data (April 9, 2023), and National Records of Scotland (NRS) mortality records (April 5, 2023). Additionally, data were linked to SARS-CoV-2 testing data (from the Electronic Communication of Surveillance in Scotland, or ECOSS: up to April 17, 2023); COVID-19 vaccinations (Turas Vaccination Management Tool and GP records: March 26, 2023), and SARS-CoV-2 virus sequencing data (April 1, 2023).

Note that for SMR01, data in the last six weeks of admissions available is unlikely to be complete, due to admissions only being added once their clinical coding was available.

Supplementary Notes

**GRIPP2 Reporting Guidelines: Short Form**

Aims

The aims of patient and public involvement (PPI) with this study were fourfold: to involve people who were eligible for/had received COVID 19 therapeutics, and the wider public in the (1) design and (2) interpretation of the project analysis; and to contribute directly to the (3) academic and (4) public dissemination of the study results, through peer-reviewed publications and summaries in plain English respectively.

Methods

This study uses routinely collected health data from the “Early Pandemic Evaluation and Enhanced Surveillance of COVID-19” (EAVE II) platform. Two patient partners (Laidlaw, Sterniczuk) were recruited in February 2022 from the EAVE II Public Advisory Group (PAG; n=15), established in November 2020, to become PPI Co-Leads for the project. The Co-Leads were invited to form a PPI Team on the basis of PPI and lived experience, knowledge of EAVE II and health data considerations, and eligibility for the treatments being studied.

The PPI Team have been involved in reviewing commentary from the wider PAG group on the initial protocol summary; designing the final analysis protocol; discussing the overall project and interpreting treatment uptake results with the PAG; interpreting treatment effectiveness results directly with the primary analyst (Tibble) and PPI Coordinator (Woolford); contributing to publication manuscripts; critically appraising analytical interpretations from a patient perspective; and writing publication summaries in plain English to share the work with public audiences. We have also co-written this Appendix with the PPI Coordinator, evaluating PPI within the project.

This work was carried out remotely, either using video-conferencing (Zoom, with minutes produced from each recording) or asynchronously via email. Co-Leads and PAG contributors were paid for time and expertise given in line with National Institute for Health and Care Research (NIHR) guidelines.

Results

Nine PAG members commented on the initial analysis plan, facilitated by a Plain Language Summary and guided questions. As well as answering the guided questions, we were encouraged to express comments, raise queries and concerns, and comment on the analysis summary wording.

There was a distinct split between general and treatment-eligible contributors in terms of their awareness, understanding and information seeking regarding COVID-19 therapeutics. This prompted us to consider more detailed research summaries for eligible patients to meet their specific needs, and signposting to mainstream media articles or explanatory resources for the non-eligible wider public who may be interested.

Polypharmacy and the risk-benefit ratio of receiving additional treatments were raised as key considerations for eligible patients. As a result of this, the analyst team included existing prescriptions of cohort patients in the analysis, and reported on possible treatment interactions, within the bounds of reporting on small patient numbers in the research.

Overall, PAG members were happy with the proposed research, but suggested several clarifications, edits to the design, and reframing of language (e.g. when discussing risk of negative outcomes). The primary analyst responded to all clarification requests, and incorporated one design suggestion which was feasible within the limits of the data available (including tests of pulmonary function).

Available data sources were then discussed by the PPI Team and primary analyst in greater detail, to further refine the protocol. This included discussion of geographical variations in hospital versus community prescribing, and in immunotherapy prescribing generally. Time between diagnosis and treatment was subsequently included in the analysis protocol, as a significant – and potentially variable – factor in patient care and outcomes.

The EAVE II PAG met to discuss the project more broadly as it progressed, providing important insights into how to communicate the study to the general public, and what questions they may have about COVID-19 therapeutics. This included clarification on the relationship between ongoing safety and effectiveness research, full or conditional market authorisations for different treatments, and whether therapeutics were designed specifically to treat COVID-19 and/or to be used in combination with each other.

Following generation of results for this study, the PPI Team interpreted the results and edited the draft manuscript. Two key areas for consideration were the number of eligible patients invited to receive treatment compared to untreated eligible patients, and significant variations in hospitalisation risk between different groups of patients treated with immunosuppressants (e.g. with/without rheumatoid arthritis or systemic lupus erythematosus). The manuscript was updated to draw attention to these results and potential limitations.

Discussion and conclusions

Despite the accelerated timeframe for this study, PPI has had a significant influence on its development, from preliminary designs through to study publication and plans for dissemination. There have been five specific changes to the analysis design and interpretation, defined by the inclusion of further patient-relevant variables, or drawing attention to results or limitations of interest from a patient perspective. Involvement has also helped to define how we will communicate the study to different public audiences, and which content we will need to explain in greater detail (e.g. treatment authorisation and mechanism of action).

Reflections

A key strength of PPI on this study has been the willingness of the primary analyst to discuss the project in detail with patient/public contributors, respond proactively to suggestions for change, and explain why certain suggestions could not be incorporated. This has resulted in improvements to the quality of the study and its benefit to patients. It has also fostered a meaningful working relationship with the PPI Team, a clear sense of where we have made changes to the project, and a record of where the available data may be limited from a patient perspective.

We felt that it was important to include a GRIPP2 annexe with this study to add to evidence about the ways PPI can contribute to data research. Involving people with different experiences of and risk from COVID 19, and eligibility for therapeutics, was crucial to capture different perspectives.

Our PPI Team have felt meaningfully included in detailed analysis development, but would have liked to see additional opportunities for involvement at a general project steering level, to better understand the overall research narrative and directions for future research. We feel that this point is important to raise with respect to PPI in big data research; what level of involvement should be expected, funded and facilitated?

Supplementary Table 1: STROBE Checklist for Cohort Studies

|  | Item No | Recommendation | Page No |
| --- | --- | --- | --- |
| **Title and abstract** | 1 | (*a*) Indicate the study’s design with a commonly used term in the title or the abstract | 1 |
|  |  | (*b*) Provide in the abstract an informative and balanced summary of what was done and what was found |  |
| Introduction | | | |
| Background/rationale | 2 | Explain the scientific background and rationale for the investigation being reported | 5 |
| Objectives | 3 | State specific objectives, including any prespecified hypotheses | 5 |
| Methods | | | |
| Study design | 4 | Present key elements of study design early in the paper | 5 |
| Setting | 5 | Describe the setting, locations, and relevant dates, including periods of recruitment, exposure, follow-up, and data collection | 5,6 |
| Participants | 6 | (*a*) Give the eligibility criteria, and the sources and methods of selection of participants. Describe methods of follow-up |  |
|  |  | (*b*) For matched studies, give matching criteria and number of exposed and unexposed | 6,7 |
| Variables | 7 | Clearly define all outcomes, exposures, predictors, potential confounders, and effect modifiers. Give diagnostic criteria, if applicable | 6,8 |
| Data sources/ measurement | 8* | For each variable of interest, give sources of data and details of methods of assessment (measurement). Describe comparability of assessment methods if there is more than one group | 5,6 |
| Bias | 9 | Describe any efforts to address potential sources of bias | 7 |
| Study size | 10 | Explain how the study size was arrived at | 6,7 |
| Quantitative variables | 11 | Explain how quantitative variables were handled in the analyses. If applicable, describe which groupings were chosen and why | 8 |
| Statistical methods | 12 | (*a*) Describe all statistical methods, including those used to control for confounding | 8 |
|  |  | (*b*) Describe any methods used to examine subgroups and interactions |  |
|  |  | (*c*) Explain how missing data were addressed |  |
|  |  | (*d*) If applicable, explain how loss to follow-up was addressed |  |
|  |  | (*e*) Describe any sensitivity analyses |  |
| Results | | |  |
| Participants | 13* | (a) Report numbers of individuals at each stage of study—eg numbers potentially eligible, examined for eligibility, confirmed eligible, included in the study, completing follow-up, and analysed | 9 |
|  |  | (b) Give reasons for non-participation at each stage |  |
|  |  | (c) Consider use of a flow diagram |  |
| Descriptive data | 14* | (a) Give characteristics of study participants (eg demographic, clinical, social) and information on exposures and potential confounders | 10,11,12 |
|  |  | (b) Indicate number of participants with missing data for each variable of interest |  |
|  |  | (c) Summarise follow-up time (eg, average and total amount) |  |
| Outcome data | 15* | Report numbers of outcome events or summary measures over time | 13,14 |

| Main results | 16 | (*a*) Give unadjusted estimates and, if applicable, confounder-adjusted estimates and their precision (eg, 95% confidence interval). Make clear which confounders were adjusted for and why they were included | 14,15 |
| --- | --- | --- | --- |
|  |  | (*b*) Report category boundaries when continuous variables were categorized |  |
|  |  | (*c*) If relevant, consider translating estimates of relative risk into absolute risk for a meaningful time period |  |
| Other analyses | 17 | Report other analyses done—eg analyses of subgroups and interactions, and sensitivity analyses | 15 |
| Discussion | | | |
| Key results | 18 | Summarise key results with reference to study objectives | 15,16 |
| Limitations | 19 | Discuss limitations of the study, taking into account sources of potential bias or imprecision. Discuss both direction and magnitude of any potential bias | 17 |
| Interpretation | 20 | Give a cautious overall interpretation of results considering objectives, limitations, multiplicity of analyses, results from similar studies, and other relevant evidence | 16,17 |
| Generalisability | 21 | Discuss the generalisability (external validity) of the study results | 16,17 |
| Other information | | | |
| Funding | 22 | Give the source of funding and the role of the funders for the present study and, if applicable, for the original study on which the present article is based | 1 |

Supplementary Table 2: Patient Comorbidities for Treatment Episodes by Medication, in the non-hospitalised Healthcare Setting (Group 3)

|  | Molnupiravir  (n=2910) | Nirmatrelvir + Ritonavir (n=5688) | Sotrovimab (n=3229) |
| --- | --- | --- | --- |
| Chemotherapy | 360 (12.4%) | 763 (13.4%) | 408 (12.6%) |
| Blood Cancer | 258 (8.9%) | 476 (8.4%) | 254 (7.9%) |
| Cirrhosis | 82 (2.8%) | 82 (1.4%) | 84 (2.6%) |
| Chronic Kidney Disease (Stage 3+) | 630 (21.6%) | 213 (3.7%) | 861 (26.7%) |
| Down Syndrome | 21 (0.7%) | 66 (1.2%) | 22 (0.7%) |
| HIV/AIDS | 30 (1.0%) | 51 (0.9%) | 16 (0.5%) |
| Immunosuppressants Prescribed | 595 (20.4%) | 644 (11.3%) | 807 (25.0%) |
| Neurological Condition | 355 (12.2%) | 1076 (18.9%) | 410 (12.7%) |
| Radiotherapy | 29 (1.0%) | 49 (0.9%) | 29 (0.9%) |
| Rheumatoid Arthritis or Systemic Lupus Erythematosus | 472 (16.2%) | 1204 (21.2%) | 526 (16.3%) |
| Respiratory Cancer | 25 (0.9%) | 28 (0.5%) | 18 (0.6%) |
| Solid Organ Transplant | 527 (18.1%) | 813 (14.3%) | 649 (201%) |
| Stem Cell Transplant | 18 (0.6%) | 32 (0.6%) | 20 (0.6%) |

Notes: The following high-risk categorised comorbidities were excluded from analyses to suppress low numbers and protect patient confidentiality: Bone Marrow Transplant, Sickle Cell Disease, and Splenectomy.

Supplementary Table 3: Treatment Episodes by Variant and Most Prevalent Strain at time of diagnosis.

| **Row / Column Total** | | **BA.1 Period**  **(**December 21, 2021, to February 28, 2022**)** | **BA.2 Period**  **(**March 1, 2022, to May 31, 2022) | **BA.5 Period**  (June 1, 2022, to September 26, 2022) |
| --- | --- | --- | --- | --- |
|  |  | **Number of treatment episodes for sequenced patients** | | |
|  |  | 1116 | 1330 | 861 |
| B.1.617.2 (Delta 1) | 63 | 63 | 0 | 0 |
| AY.4.2 (Delta 2) | 29 | 29 | 0 | 0 |
| BA.1 (Omicron 1) | 965 | 802 | 132 | 37 |
| BA.2 (Omicron 2) | 1516 | 222 | 1186 | 99 |
| BA.4 (Omicron 4) | 96 | 0 | 2 | 95 |
| BA.5 (Omicron 5) | 640 | 0 | 10 | 630 |

Supplementary Table 4: Treatment Episodes by Most Prevalent Strain at time of diagnosis and Medication

| **Medication** | **BA.1 Period (n=3693)** | **BA.2 Period (n=5875)** | **BA.5 Period (n=5064)** |
| --- | --- | --- | --- |
| Combination | 114 (3.1%) | 79 (1.3%) | 53 (1.0%) |
| Molnupiravir | 1037 (28.1%) | 1017 (17.3%) | 884 (17.5%) |
| Nirmatrelvir + Ritonavir | 331 (9.0%) | 3043 (51.8%) | 2818 (55.6%) |
| Remdesivir | 139 (3.8%) | 392 (6.7%) | 418 (8.3%) |
| Sarilumab | 252 (6.8%) | 27 (0.5%) | 14 (0.3%) |
| Sotrovimab | 1704 (46.1%) | 1211 (20.6%) | 807 (15.9%) |
| Tocilizumab | 116 (3.1%) | 106 (1.8%) | 70 (1.4%) |
